# Therapeutic role of respiratory exercise in patients with tuberculous pleurisy

**DOI:** 10.1515/med-2024-1057

**Published:** 2024-11-07

**Authors:** ZengXia Ma, GuiMei Liu, GaoFeng Qiao, ChangMing Shen, Cheng Wang

**Affiliations:** Department of Respiratory and Critical Care Medicine, Shandong Public Health Clinical Center, Shandong University, Shandong, 250013, China; Department of Tuberculosis, Shandong Public Health Clinical Center, Shandong University, Shandong, 250013, China; Department of Thoracic Surgery, Shandong Public Health Clinical Center, Shandong University, Shandong, 250013, China

**Keywords:** respiratory exercise, tuberculous pleurisy, lung function, pleural hypertrophy

## Abstract

**Objective:**

To observe the efficacy of respiratory exercise in patients with tuberculous pleurisy (TBP).

**Methods:**

A randomized controlled study was conducted including 146 patients diagnosed with TBP and undergoing pleural effusion drainage in Shandong Public Health Clinical Center from June 2020 to December 2022, and the patients were randomly divided into the control group and the respiratory exercise observation group. Pleural effusion drainage time, the difference of pulmonary function, and the degree of pleural hypertrophy between the two groups at 1 and 3 months after treatment were studied.

**Results:**

Compared with the control group, the pleural effusion drainage time of the observation group was shortened, and there was no significant difference between the two groups in terms of lung function and the degree of pleural hypertrophy at 1 month after treatment, while the lung function indexes and the degree of pleural hypertrophy of the observation group were significantly improved compared with that of the control group at 3 months after treatment.

**Conclusion:**

Respiratory exercise can shorten the drainage time of effusion in patients with TBP, and help to improve lung function and alleviate pleural hypertrophy adhesion.

## 1 Introduction

Tuberculous pleurisy (TBP) is the most common form of extrapulmonary tuberculosis, caused by *Mycobacterium tuberculosis* and its metabolites which are transmitted to the pleural cavity via blood or lymph or direct invasion of the pleura by tuberculosis foci [1]. Improper treatment may result in complications such as pyothorax, pleural hypertrophic adhesions, and liquid pneumothorax [2], and in severe cases, could cause thoracic deformities, which can affect the patients’ pulmonary function and life. Respiratory exercise is mainly used to improve lung function in patients with chronic lung disease and after thoracic and abdominal surgery [3–5], and its efficacy in patients with TBP is not clear yet. In the present study, we observed the effects of respiratory function exercises on the pleural fluid drainage time, lung function, and pleural hypertrophy in patients with tuberculous pleural effusion, which improved the patients’ quality of life and confidence in treatment by improving their prognosis.

## 2 Data and methods

### 2.1 Patient inclusion and exclusion criteria

Inclusion criteria included: (a) aged 18–68 years old, (b) meeting the diagnostic criteria standard of the People’s Republic of China, “WS 288-2017 Diagnosis of Tuberculosis,” and all received standardized anti-tuberculosis treatment and drainage of pleural effusion during the hospitalization period, with a minimum of 3 months follow-up for each patient, (c) being able to use the respiratory trainer correctly and (d) willing to join and cooperate with the study, all of them signed an informed consent form, which was approved by the hospital’s ethics committee.

Exclusion criteria included: combination of other pulmonary diseases and serious heart, liver and kidney diseases and other comorbidities, inability to tolerate anti-TB treatment, and infection at or near the puncture site were excluded.

### 2.2 General data of patients

A total of 146 patients diagnosed with TBP and underwent pleural effusion drainage in Shandong Province Public Health Clinical Center from June 2020 to December 2022 were collected as the study subjects. The random number table method was used for grouping, 72 cases in the control group, 47 men and 25 women, with an average age of 31.95 ± 9.98 years old; while 74 cases in the observation group with an average age 32.67 ± 11.57 years old, 48 men and 26 women. The difference of gender, age and other information of the two groups is not statistically significant (*P* > 0.05).

### 2.3 Methods

The control group was given standardized anti-tuberculosis treatment, ultrasound localization, or guided thoracic closed drainage; urokinase was injected into thoracic when adhesion was obvious. The drainage was removed when the pleural effusion could not continue to be pumped out, or no obvious effusion was proved after review of ultrasound. Respiratory exercise was adopted additionally in the observation group on the basis of the control group. Respiratory trainer (5,000 mL capacity type) was used for respiratory training which was easy to carry and to operate both in hospital and at home. The training time was set to 20–30 min, three times per day. The degree of pleural hypertrophy is assessed by chest CT examination, through measuring the most obvious part of pleural thickening, which was evaluated by two senior radiologists and respiratory doctors.

### 2.4 Clinical evaluation

Compare the differences in the drainage time of pleural effusion, lung function including forced expiratory volume in one second (FEV1), forced vital capacity (FVC), FEV1/FVC, diffusing capacity for carbon monoxide (DLCO), and pleural hypertrophy degree between the two groups at 1 and 3 months from treatment.

### 2.5 Statistical treatment

All data were expressed as *x* ± *s*. Statistical treatment was performed using the SPSS20.0 statistical software package, and the *t*-test was used for the comparison of the measurement data of the two groups.


**Ethical approval:** This study was approved by the Ethics Committee of Shandong Public Health Clinical Center.

## 3 Results

### 3.1 Drainage time of pleural effusion in the two groups

Comparing the time of pleural drainage in the two groups, it can be seen that the drainage time of 72 patients in the control group was 17.8 ± 7.9 days, while the drainage time of 72 patients in the observation group was 15.3 ± 6.3 days, which is statistically different (*p* = 0.036), suggesting that respiratory function exercise can shorten the drainage time of pleural effusion.

### 3.2 Changes in pulmonary function indexes of the two groups (Table 1)

**Table 1:** Comparison of lung function parameters of the two groups

	0 month				First month				Third month			
	FEV1 (L)	FVC (L)	FEV1/FVC (%)	DLCO	FEV1 (L)	FVC (L)	FEV1/FVC (%)	DLCO	FEV1 (L)	FVC(L)	FEV1/FVC (%)	DLCO
The observation group (*n* = 74)	2.02 ± 0.47	2.33 ± 0.71	86.11 ± 7.75	5.24 ± 0.45	2.51 ± 0.75	2.91 ± 0.81	86.25 ± 8.99	7.18 ± 0.68	2.90 ± 0.84	3.49 ± 0.89	83.09 ± 7.36	8.95 ± 0.64
The control group (*n* = 72)	1.94 ± 0.61	2.21 ± 0.85	87.92 ± 9.61	5.13 ± 0.60	2.42 ± 0.61	2.73 ± 0.89	88.64 ± 9.12	6.99 ± 0.72	2.76 ± 0.88	3.18 ± 0.74	86.79 ± 8.11	8.65 ± 0.73
*P*	0.3772	0.3568	0.2132	0.2133	0.4284	0.2031	0.1130	0.1033	0.3270	0.0238	0.0045	0.0091

Lung function examination found that the difference of lung function indexes between the two groups was not statistically significant at first month, while FEV1 in the observation group had a tendency to increase compared with that of the control group at third month, but the difference was not statistically significant, FVC and DLCO in the observation group increased compared with that of the control group and the difference was statistically significant, and FEV1/FVC in the observation group decreased compared with that of the control group and the difference was statistically significant.

### 3.3 Changes in pleural thickness in the two groups (Table 2)

**Table 2:** Changes in pleural thickness in the two groups

	First month	Third month	*P*
The observation group (*n* = 74)	0.34 ± 0.06	0.24 ± 0.08	<0.0001
The control group (*n* = 72)	0.33 ± 0.08	0.27 ± 0.07	<0.0001
*P*	0.3934	0.0173	

**Figure 1:**
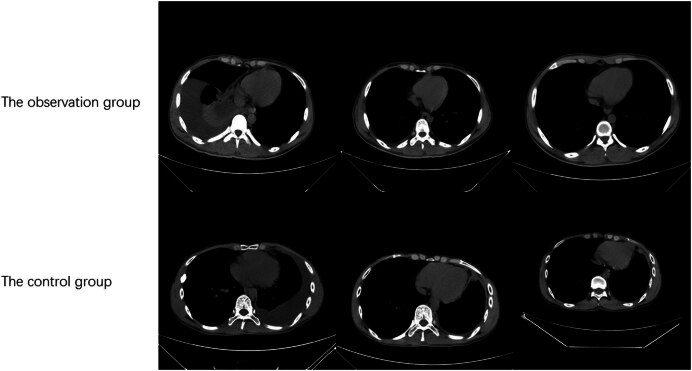
Changes of CT images of the two groups.

With the prolongation of the treatment time, the pleural thickness gradually became smaller (Figure 1), and the pleural thickness at third month was significantly smaller than that at first month. The difference in pleural thickness between the two groups was not statistically significant at first month, and pleural hypertrophy was reduced in the observation group compared with the control group at third month and the difference was statistically significant.

## 4 Discussion

TBP is one of the most common extrapulmonary tuberculosis, with an incidence rate of about 54.8% among all patients with pleurisy, which is in the first place [6]. TBP occurs as a result of direct invasion of the pleura by tuberculosis foci or transmission of *Mycobacterium tuberculosis* and its metabolites to the pleural cavity by blood or lymph. Tuberculous pleural effusion is rich in proteins, inflammatory cells, inflammatory factors, and various cytokines, while the interaction of the above factors promotes the formation of pleural granulation tissue which leads to pleural thickening, adhesion, and the formation of encapsulated pleural effusion [7]. Impaired blood and lymphatic reflux in the pleura result in localized ischemia, hypoxia, and inflammatory reaction, further leading to localized pleural hyperplasia and hypertrophy adhesion. In a review of post-tuberculosis lung disease, 50% of chest radiograph participants and 60% of CT examination participants reported a mild pleural thickening of >2 mm, and a residual pleural thickening of >10 mm was found in 20–46% of patients [8], and we observed that the average pleural thickness of the control and observation groups of patients with TBP at first month after treatment were both greater than the third month, with a non-statistically significant difference, which indicate that pleural hypertrophy is very common in the early stage of TBP. The observation group with the addition of respiratory exercise, the degree of pleural hypertrophy 3 months after treatment reduced compared with the control group, and the difference is statistically significant, indicating that prolonged adherence to respiratory exercise can reduce the pleural hypertrophic adhesion. However, there is no research literature about “respiratory exercise” and “TBP” and “pleural hypertrophy.”

Pleural thickening can limit lung recuperation, increase elastic resistance, decrease lung and chest wall compliance, decrease lung function indexes such as vital capacity, total lung capacity (TLC), one-second exertion expiratory volume (FEV1), and residual volume of air (RV), while the ratio of exertion lung capacity (FEV1/FVC) and RV/TLC showed a tendency to increase, and restrictive ventilation dysfunction appeared eventually [9]. In severe cases, the diffusion function decreases, resulting in varying degrees of hypoxia and carbon dioxide retention in patients. The prolonged presence of clinical symptoms in patients and their own psychological factors also have a certain impact on pulmonary function. We observed that patients with TBP showed restrictive ventilatory dysfunction and decreased ventilatory function in the early stage, which was considered to be related to pleural thickening, abnormal local blood and lymphatic return, and imbalance in the ratio of ventilation and blood flow.

Patients with TBP who are not treated in time may develop pleural hypertrophy of varying degrees, which in severe cases can have lasting effects on pulmonary function and the patient’s later life. The treatment of tuberculous pleural effusion mainly includes [10] pleural effusion drainage, anti-tuberculosis drug therapy, short-term application of glucocorticoids, localized use of thoracic fibrinolytics, and thoracoscopic and surgical interventions in severe cases; however, it is still difficult to recover their lung function to a satisfactory degree for some patients [11]. Respiratory exercise is not only well used in international guidelines for the management of COPD and other lung diseases, but also recommended in tuberculosis guidelines [12]. Various studies have suggested that patients with pleural effusion should be included in respiratory physiotherapy programs as early as possible. Respiratory exercise can increase the static volume of the lungs, also improve the efficiency of the inspiratory muscles [13], which can promote the drainage of fluid and help patients increase their compliance with treatment and improve prognosis [14].

In the present study, a total of 146 patients with tuberculous pleural effusion were included, with a non-significant difference in the baseline level. On the basis of conventional treatment, we added respiratory exercise to the observation group, and found that the drainage time of pleural effusion in the respiratory exercise group was shorter than that in the control group (15.3 ± 6.3), and the difference was statistically significant, which indicates that respiratory exercise promotes the drainage of effusion, shortens the drainage time of pleural fluid, and reduces the chance of chest infection. There was no significant difference between the control group and the observation group in terms of pulmonary function indexes at 1 month after treatment. FEV1 of the observation group increased compared with that of the control group at 3 months after treatment, though the difference was not statistically significant. FVC and DLCO of the observation group increased compared with that of the control group with a statistically significant difference, while FEV1/FVC of the observation group significantly decreased compared with that of the control group at the same time. FEV1, FVC, and DLCO were all significantly higher (*P* < 0.05), FEV1/FVC was significantly lower in the observation group than that at first month (*P* < 0.05), while FEV1/FVC in the control group was lower than that at third month with a non-statistically significant difference. The above data indicate that respiratory exercise can promote the recovery of lung function, improve ventilation and gas exchange, which is comparable to that observed by Valenza-Demet et al. [15].

Respiratory exercise helps to reduce the duration of chest drainage and reduce pleural hypertrophy, improve pulmonary ventilation, and air exchange function. Its possible reasons include [16–18]: (a) respiratory exercise can increase the negative pressure in the pleural cavity of the patient and expand the thorax to avoid alveolar atrophy and collapse, and promote drainage; (b) can improve the function of respiratory muscles, increase the maximum ventilation, and enhance the exercise endurance; (c) improve the local blood circulation and oxygen supply, reduce the inflammatory reaction of the pleura locally, and reduce the occurrence of pleural thickening and adhesion; and (d) the bedside respiratory training by the medical staff increases the patients’ enthusiasm and confidence in treatment, which is conducive to the improvement of lung function. However, the sample size of this observation was small, no subgroup analysis was conducted, and the whole follow-up was not conducted, so the above problems need to be further solved in the future.

In conclusion, respiratory exercise should be added to the routine treatment of patients in the early stage, which can shorten the time of chest drainage, reduce the chances of chest infection, effectively improve the lung function of the patients, reduce the pleural hypertrophy and adhesion, and increase the patients’ confidence in the treatment, quality of life, and the degree of cooperation, which is worthy of popularization and application in the clinic.
